# Evaluation of a 'virtual' approach to commissioning health research

**DOI:** 10.1186/1478-4505-4-9

**Published:** 2006-10-18

**Authors:** Christine A McCourt, Philip A Morgan, Penny Youll

**Affiliations:** 1 Faculty of Health and Human Sciences, Thames Valley University, 32-38 Uxbridge Road, London W5 2BS, UK; 2 Tavistock Institute, 30 Tabernacle St. London, EC2A 4UE, UK

## Abstract

**Background:**

The objective of this study was to evaluate the implementation of a 'virtual' (computer-mediated) approach to health research commissioning. This had been introduced experimentally in a DOH programme – the 'Health of Londoners Programme' – in order to assess whether is could enhance the accessibility, transparency and effectiveness of commissioning health research. The study described here was commissioned to evaluate this novel approach, addressing these key questions.

**Methods:**

A naturalistic-experimental approach was combined with principles of action research. The different commissioning groups within the programme were randomly allocated to either the traditional face-to-face mode or the novel 'virtual' mode. Mainly qualitative data were gathered including observation of all (virtual and face-to-face) commissioning meetings; semi-structured interviews with a purposive sample of participants (n = 32/66); structured questionnaires and interviews with lead researchers of early commissioned projects. All members of the commissioning groups were invited to participate in collaborative enquiry groups which participated actively in the analysis process.

**Results:**

The virtual process functioned as intended, reaching timely and relatively transparent decisions that participants had confidence in. Despite the potential for greater access using a virtual approach, few differences were found in practice. Key advantages included physical access, a more flexible and extended time period for discussion, reflection and information gathering and a more transparent decision-making process. Key challenges were the reduction of social cues available in a computer-mediated medium that require novel ways of ensuring appropriate dialogue, feedback and interaction. However, in both modes, the process was influenced by a range of factors and was not technology driven.

**Conclusion:**

There is potential for using computer-mediated communication within the research commissioning process. This may enhance access, effectiveness and transparency of decision-making but further development is needed for this to be fully realised, including attention to process as well as the computer-mediated medium.

## Background

This article describes the evaluation of a novel process of commissioning of health research, using computer-mediated communication (CMC). A website was designed and piloted to enable committees to meet 'virtually' to agree priorities and commission projects. An open recruitment process was also used, including adverts for membership in the popular press. This 'naturalistic experiment' was conceived by the Department of Health with the aims of including a wider constituency in the research process, and so sits within recent policy and practice emphasis within the health service of increasing inclusion in policy, research and practice. It was also intended to enhance the effectiveness and transparency of the process, to contribute to the quality of health research. The authors were commissioned by the UK Department of Health to evaluate the effects of this experiment. The aims agreed for the study were to assess the practical workings of this new approach, its feasibility and whether it may have the intended benefits of enhancing the accessibility, transparency or quality of the research commissioning process. Taking into account the nature of the programme, and the early stage of experimentation with this approach to research commissioning, we opted to use an action research approach, to ensure learning and feedback within the process. The random allocation of different groups to the novel virtual approach or the traditional face-to-face approach enabled us to combine this with a naturalistic-experimental approach using comparison of the process and experiences of the different commissioning groups.

This paper reports on our overall evaluation and a following paper will discuss the conceptual issues raised relating to CMC in greater depth.

The UK Department of Health in the 1990s commissioned research on a range of health related topics centrally, in particular priority areas and through its regional offices. During the decade there were shifts towards a more strategic approach – designed to meet specific health objectives – and a more inclusive approach, intended to involve practitioners and users of health services more fully in the research process. Greater attention was also focused on the effectiveness of health research and how far it was meeting the needs of the service and of the nation's health. The project studied here sits within that context of development. Traditionally, health research at central and regional level has been planned and commissioned by formal committees, meeting face to face and involving mainly health policy and research experts, supported by anonymous expert peer review of proposals. The 'virtual commissioning' project we studied represented an attempt to move the traditional process towards one that would be more transparent, more effective and more inclusive.

Given the early stage of the work, we focus on the process, as observed and as perceived by the participants, in particular the nature of online, asynchronous communication within relatively formal, task-focused groups, and examine some early indicators of outcomes. Key themes emerging included access to participation, interactivity within formal groups (both virtual and face-to-face) the role of language in this, and the uses of time. We also examine indicators of the quality of commissioning resulting from this approach. The issues arising in this study may provide reflection on interaction within formal groups in a range of settings and support the argument that the impact of different media are socially situated, not technically dependent. Nonetheless, the evaluation highlights issues that need to be considered in designing an environment and process for 'virtual' committees.

### Relevant literature

A comprehensive review of the literature was undertaken using CINAHL, Medline and Sociological Abstracts as well as a number of education and management databases. The criteria for inclusion focussed on articles covering research commissioning, consumer involvement and widening participation in research commissioning as well as the general effects of using CMC and its more specific influence on communication, participation and decision making. This review of the literature provides an overview of the impact of widening participation in research commissioning and the effects of using computer mediated communication on this process.

#### Research commissioning and impact

Despite a growing interest in the effectiveness of research and its impact on practice, we found very little work that directly focused on the process of commissioning and its impact on the quality of research. However, potential payback from health research has been operationalised into relevant aspects [1]. Involvement in research can result in, for example, increased research awareness, organisational development, political or administrative benefits, improved resource allocation and other outcomes not directly associated with the overall research findings or their utilisation. This work also indicated that the process of commissioning has an impact on the focus, conduct and monitoring of research. Research has also been conducted on NHS research payback using a bibliometric approach [2]. However, this addressed the outputs of commissioned projects rather than the process of commissioning.

Failure to effectively communicate health research, suggest Kuruvilla & Mays (2005) [3], is a critical problem that can only be tackled by recognising that science is a social enterprise and that as such has social implications. They argue for opportunities for diverse groups to share their views and experience to ensure a range of perspectives and resources can be integrated to meet complex health challenges. Clearly, involving consumers in the research and development agenda setting for the NHS is an increasing priority and recent work has concluded that involving consumers effectively requires appropriate skills, resources, time, good communication and targeted support, including training [4].

Research undertaken in other formal contexts could be applied to questions around decision-making and committee processes within research commissioning. Studies such as Tomlinson's [5] indicate that decision-making by committees may not work in the ways that official policy assumes. For example, to avoid open conflict, important aspects often take place outside the committee room. The gaps revealed in our review highlight the degree to which the *established *processes of research commissioning remain relatively taken-for-granted and unevaluated.

#### Computer-mediated communication (CMC)

##### Media effects

The literature on CMC uses two key perspectives to understand the interaction and decision-making processes of virtual groups – media effects and relational issues. The main theory of media effects – the *reduced social context cues model *– refers to the filtering out of (visual and audible) cues in CMC [6]. These may be markers of members' identity and status as well as indicators of response. Such filtering effects may constrain or even distort communication [7]. The reduced social context cues model has received considerable criticism recently, particularly since the model is mainly derived from experimental studies using one-shot, zero history groups (usually students) working on time-limited tasks using only synchronous conferencing [8]. The stark view of CMC reported by such studies is contradictory to those found in more longitudinal field research.

#### Relational issues

The *relational *stance [9] is based on the premise that what happens in a virtual system is not the product of the technology alone but of social relationships, and other contextual factors, and the way these interface with the technology [10].

This perspective does suggest, however, that CMC groups may take four to five times longer to communicate than face-to-face groups due mainly to the reduction of social context cues and the relatively more complex and time consuming nature of the textual medium [9] and this is accompanied by, and related to, interpersonal development and improved group cohesiveness [11].

The role of Group factors including trust, particularly the notion of *swift trust *[12] have also been discussed with respect to CMC. Ishaya and Macaulay report a link between trust and performance and that developing and maintaining trust is contingent on a range of actions that contribute to the cohesion of the group. Chief among these is commitment [10] and persistence [13] and these have been linked to the level of activity and the richness of the virtual environment. A sense of team, cohesion and humour have been found necessary for successful virtual group work just as they are in the face-to-face situation and are enhanced by frequent, prompt and proactive, rather than reactive, communication [14]. While these factors seem equally relevant to face-to-face groups, it is possible that the nature of the medium means that such features of the environment have a particular importance for virtual groups.

Other key group factors include the selection and use of multiple media to support a dynamic and rich virtual environment [15]. Equally important in encouraging more relationally positive interaction is the anticipation of future interaction [9]. The importance of preparation and training, particularly for the role of moderator or chairperson, and production of protocols, ground rules and guidance for on-line etiquette have also been shown to be important for effective participation. Such preparation and training is also linked to the promotion of a positive and busy environment and a satisfying on-line experience [16] and the reduction of uncertainty [14]. As noted, much of the existing research has employed experimental rather than 'real-life' groups, often using students as subjects or has looked at less formal or task-focused groups.

#### Achieving consensus and making decisions

Achieving consensus and making decisions in CM groups also raises questions about the place of conflict in CM decision-making and the question of status and equality. Early studies reported that CMC has the potential to promote greater equality than face-to-face groups [17]. However, in organisational contexts where anonymity is not appropriate or possible, studies suggest the opposite to be the case, with status and power being reinforced [18]. Mantovani [19] points out that even if a low-status member contributes equally, this does not mean that their contribution has been given equal weight to those of high status members. He reminds us that unlike the anonymity of experimental settings, that gave rise to the notion of status equalisation, users of CMC in organisational settings are often very aware of status differentials and act accordingly.

#### Consumer involvement in research

There is a growing literature on consumer involvement in research and policy, which is not reviewed in detail here. A series of recent papers have made clear the commitment to the principles of involvement [20,21], and have begun to address practice issues [22,23]. The importance of involving consumers at all stages of development, through from setting priorities and asking research questions to critically reviewing research and policy has been noted [24,25], and the Programme evaluated here was clearly responding to such advice. Reasons for involving consumers have also been described [25,26] and include both moral and functional considerations. Both were implicit in the aims of using a virtual approach in this Programme – to enhance the equity as well as effectiveness of research. However, examples of good practice in health research remain limited [27,28] and there is a need to develop mechanisms and processes to ensure that the role of consumers in health research is clear and well supported.

## Context, design and methods

### The Programme

The inception of a new Research Programme to address London's health was taken by the UK Department of Health as an opportunity to develop and evaluate new approaches to commissioning that seek to move towards a more inclusive, effective and accountable process. This Programme, which was the subject of our evaluation, aimed to address issues known to affect the health of Londoners such as high levels of deprivation and health inequality, mobility and ethnic diversity. [29-31] through setting up six commissioning groups to work on different themes relevant to this. The Deprtment of Health's aims of experimenting with modes of commissioning in this Programme were broad, including organisational, representational, educational, quality and ethical considerations.

The first of the six Commissioning Groups within the Programme was used by the Department of Health Research and Development Office to pilot a novel, 'virtual' commissioning process. This initial pilot was evaluated by the authors and the resulting report recommended further development [32]. Following this, the virtual approach was amended and rolled out across the remaining five commissioning groups of the Programme, each dealing with a particular theme. Each group was randomly assigned to either a 'virtual' or a traditional 'face-to-face' meeting approach and a further evaluation, which we report here, was planned. Several commissioning group members had previously participated in the pilot virtual group. They were from different backgrounds and had varying levels of commissioning experience, and they were allocated to different groups, giving a useful mix of experiences to draw on.

Traditionally, health research commissioning has used face-to-face committees, mainly involving a range of people with relevant research expertise. Such committees may be long term, or time-limited and focused around specific programmes. The commissioning groups evaluated here were short term since they were formed specifically for the Health of Londoners Programme and their main tasks were to set priorities for research in their theme areas as decided by the Department of Health, and to discuss and agree on which research proposals should be funded. In most respects, apart from open advertising and the virtual 'experiment', this followed a conventional health research funding process. Table 1. sets out the process, and the respective roles of the R&D office and the commissioning groups. Although it describes this particular programme, the process is typical of health research commissioning, apart from the IT aspects of using a virtual mode.

**Table 1:** Sequence of commissioning activities and the roles of DoH R&D officers and commissioning groups within the Health of Londoners Programme

**Stages**	**NHS R&D roles**	**Commissioning group roles**
**1: Needs assessment** **Project specification**	HoL objectives and research areas identified and prioritised; Process specified; Commissioning group members recruited/selected	Interested people respond to adverts
**2: Preparation**	Infrastructures: website, technology and Programme support; IT Training; Programme orientation meeting; Research awareness session	Knowledge & expertise Interests Attendance at induction and preparation meetings
**3: Processes** **Phase 1 – agreeing priority topics for research** **Phase 2 – commissioning projects from proposals submitted under the topics chosen**	Facilitation and information support; Develop vignettes, involving chairs of commissioning groups; Develop research briefs (researchers bid competitively, submitting written proposals); Research contracts negotiated and agreed	Web-based participation or meeting attendance: Discussion of research topics; Read external peer reviewer reports; Scrutiny of research proposals & recommendations on selection
**4: Primary outputs**	Continuing monitoring of commissioned projects Research reports Dissemination strategy	No further involvement; (Group is short-term)

A 'virtual' group does not meet physically in a room on particular dates but via a specially developed website, that can be accessed by password from any computer with internet facilities. The process remains time limited but allows a more extended discussion period (in this case, nineteen days for each Phase as compared to a three-hour meeting for each in the face-to-face mode) as well as greater physical access. The process in this Programme followed two key Phases – agreeing research priorities and reviewing and selection of proposals – each of which was concluded by anonymous voting, using a specially designed screen in the virtual mode (see summary in tables 2 &3) as compared to the expection of reaching general consensus in the face-to-face mode. The website opened with a log-in page followed by a 'virtual meeting table' with pictures and short biographies of members, before proceeding on to the business pages of the meeting. In addition to a general introductory event and a half-day research training session for the whole programme, virtual group participants were also offered an induction to using the website.

**Table 2:** Phase 1 virtual meeting – proposing and agreeing topic areas

**Days 1–2 (Mon – Tue)**	Introduction. Potential Topic Areas put forward, maximum one per member
**Days 3–9 (Wed – Tue)**	Discussion phase: exploration of Potential Topic Areas
**Day 10 (Wed)**	Chair prepares summaries
**Days 11–14 (Thu – Sun)**	Voting. Members vote for their preferred three Topic Areas
**Day 15–16 (Mon – Tue)**	Vignettes prepared by project team
**Days 17–19 (Wed – Fri)**	Members view vignettes and make comments

**Table 3:** Phase 2 virtual meeting – discussion and selection of research proposals

**Pre day 1**	Members receive hard copies of proposals relevant to their sub-committee
**Days 1–7**	Discussion at sub-committee level (online)
**Day 8**	Sub-committee Chairs prepare brief statements for General Forum. Remaining proposals needed for General Forum couriered to members.
**Days 9–15**	Discussion at General Forum level (online)
**Days 16–17**	Chair prepares Summary of discussion (including peer reviewer comments) and Options Portfolio
**Days 18–19**	Members vote on options (using a special online voting screen)

Source: Health of London Programme website

### The Aims of the Evaluation

Building on the Programme's stated objectives and the findings of the pilot study key questions were:

• Does the new approach facilitate wider participation, and if so, how?

• Does the greater transparency and 'auditability' afforded by CMC contribute to effectiveness and equity in research funding?

• Did the approach facilitate a full and focused discussion, prioritisation and selection of relevant themes?

• Could the virtual process, including the bespoke web-site, be developed further to maximise accessibility and effectiveness of use?

### Design and methods

Due to the complexity of the process being evaluated, an action research approach – intended to ensure learning and feedback within the process – was combined with a naturalistic-experimental approach [34-36]. The commissioning groups had been randomly assigned by the Programme team to work through face-to-face or 'virtual' meetings to enable comparison of experience. The small scale and early stage of the work meant that the evaluation would need to focus primarily on the experiences and perceptions of participants, although the processes would be observed and the outputs of the early work commissioned considered. Additionally, there is no established basis for identifying relationships between inputs, processes, outputs and outcomes for health research and outcomes could only be fully considered over a far longer term.

A range of methods was used within this framework including interviews, observation, collaborative enquiry groups and questionnaires. Relevant policy and committee documents and papers were also reviewed. This included statements of aims and objectives by which the process and outcomes could be evaluated.

Semi-structured interviews were conducted with a purposive fifty per cent sample of participants across the five groups, using a topic guide amended from our earlier pilot study [32]. Sampling was intended to ensure a mix of work (or consumer) roles and backgrounds, prior experience of research commissioning and to include those who did and did not participate, evenly across the theme groups. Five of these interviews were conducted by telephone, to suit the preferences of members, particularly those who had not participated actively. The remainder were conducted at a venue chosen by the member and were normally tape recorded, with permission. Informal interviews with the three Regional R&D Officers who were most closely involved with the Programme were used to explore intentions and perceptions of the virtual commissioning process.

Collaborative enquiry (CE) groups were planned to include committee members, chairpersons and Programme managers. Initially two groups were convened, one for each model, and participants were then invited to meet as an overall group. All participants were invited to take part in the CE groups.

Since the main evaluation could only follow the early process of commissioning, those projects commissioned in the previous year by the pilot virtual commissioning group were followed up to provide some initial indicators of research outputs and potential outcomes. Structured questionnaires focused on the progress of the research and its outputs, based on the model of 'research payback' (see table 4), were sent to the principal investigators of the four projects commissioned. These were followed by semi-structured face-to-face (and one telephone) interviews to explore the responses in more depth.

**Table 4:** Model of research payback

**Categories of Payback **(amended from [1])

a. Knowledge

b. Research benefits
- better targeting of future research
- development of research skills, personnel and overall capacity
- critical capability to utilise appropriately existing research

c. Political and administrative benefits
- improved information bases on which to take political and executive decisions
- other political benefits from undertaking research

d. Health and social service sector benefits
- cost reduction in delivery of existing services
- qualitative improvements in the process of service delivery
- increased effectiveness of services e.g. increased health or social welfare
- equity e.g. improved allocation of resources at an area level, better targeting and accessibility

e. Broader economic benefits
- wider economic benefits from commercial exploitation of innovations arising from R&D
- economic benefits from a healthy workforce and reduction in working days lost

This model, used in the study, was amended from the payback model developed by the Health Economics Research Group, Brunel University [1].

All participants were sent information sheets on the study and were given time to consider their participation. Confidentiality of all interview comments and observations was protected and all group participants were asked to agree ethical ground-rules including confidentiality and respect for each others' contributions.

#### Response

Following sampling, thirty-five of the sixty-six members were approached for interview and thirty-two interviews were conducted. Only two members declined, both in groups that did not complete Phase Two meetings following unexpected withdrawal of Programme funding due to DoH re-structuring. The three Collaborative Enquiry meetings were attended by six, nine and twelve members respectively. These were spread across the different theme groups, and included members who described themselves as practitioners, consumers and in academic or policy roles, often with a mix of these identities. Written, telephoned or e-mailed comments were also received from seven members who were unable to attend, who were also spread across the theme groups. Our work was limited in practice by the later than planned commissioning of this study and the early closure of the Programme. As a result we were only able to observe both phases of the process for one commissioning group. This limited the capacity for comparison of the process in the different groups.

#### Analysis

Different analytic approaches were used as relevant to the data collection methods, combining principles of grounded theory [36] with more structured techniques [38]. Open coding of interview and observation transcripts was used to identify emergent themes. More structured data produced from our observations of the commissioning groups (such as number and length of contributions, at different stages of the process) were logged onto charts to produce structured summaries of the process for each mode. Face-to-face groups were recorded by written notes, whereas the website for the virtual groups generated electronic transcripts of all the discussions. The themes and summaries produced by this analysis formed the basis for discussion in the collaborative enquiry groups (one for each mode). This discussion generated additional data as well as providing further analysis and checking of the themes. These were then mapped to a framework of criteria developed from the findings of our earlier pilot study [32]. This formed the basis of a discussion paper circulated to the members before a final collaborative enquiry meeting bringing together members in face-to-face and virtual groups to discuss the themes further and compare their experiences directly.

## Results and Discussion

### Orientation, support and facilitation

Although initial preparation meetings were organised and training provided (see table 1) members expressed disappointment with the level of orientation to the principles and process of commissioning. In particular, it was noted that group chairpersons had not been nominated at this stage and so could not facilitate an initial bringing together and orientation of each theme group at the introductory event for all the Programme's commissioning groups. As discussed in the literature, such issues have been shown to be important for participation in virtual group participation [14-16]. Our experience suggests they are also important for face-to-face group activity, but may be particularly so for a virtual approach because the setting of group norms of participation help prevent uncertainty and the potential for disruption that could result. These points are discussed below under the theme of interactivity.

In both cases, the role of the chairperson was crucial to the interaction needed to 'get the work done'. In the virtual mode, facilitation by responding encouragingly to all comments posted, seeking, summarising and providing background information, making links, introducing social-communicative styles, were extremely important for achieving a high level of dialogue to underpin the decision making.

It is different being a Chair of a web-based meeting than a face-to-face meeting. But it is the same, I think it is the same set of skills, it is the essential skills...but they need some enhancement (interview with chairperson, virtual group)

This finding echoes those of Gunawardena [16] on the role of the chair in distance education. Equally, the creation of a rich environment through the use of multi-media, including the telephone, is seen as important [15]. However, with no initial discussion of procedures and 'ground-rules', members appeared to individually develop rules for their own conduct that minimised interaction. Such uncertainties about appropriate ways to interact in the virtual mode may have added to the narrowing of range of cues in inhibiting communication, discussed below.

### Access and equity

The view that using CMC would enhance access to participation, and thereby equity of the process, was implicit in the aims of the Programme and this emerged as a strong theme in the data, but with greater complexity. Access may work on a number of levels including physical, sensory, linguistic, knowledge, confidence and ease of participation.

These issues applied to both modes – although physical access was clearly easier in CMC, since members could participate from any venue with computer and internet facilities, access to IT skills and facilities were recognised as a potential barrier. Overall, the analysis showed that although CMC has the potential to increase access to participation this does not automatically enhance participation of groups traditionally less involved in research commissioning.

Profiling of participants showed that while backgrounds were varied, few members identified themselves primarily as health service 'consumers' and the majority of those who described themselves as consumers, practitioners or retired had some experience of research or policy related work. This suggests that neither the open advertising for membership, nor the use of a virtual approach directly increased the number of non-research professional participants. Lack of records meant that we were unable to determine whether few had shown any interest in the adverts for members, or whether interested non-researchers were deterred by the tone and presentation of documents sent out to enquirers – which were thought by members to be rather technical rather than 'user-friendly' packages.

Of the virtual groups, one had no primarily 'consumer' members and the other had two, one of whom made only one contribution – to suggest a topic at the beginning of Phase One – while the other had the highest level of contributions in the group, despite being the only member without a computer, relying on public library access. The reflections of the first suggested that he did not feel clear of his role or enabled to participate effectively:

I think the discussion was not broad enough. I mean it did not take into account the different backgrounds of the group... I was not comfortable or sure of myself. I did not feel I contributed as I could have if I was in amongst other people (consumer member, virtual group)

Additionally, our observations of the discussion suggested that limited and slow online response may have been discouraging for a person without prior experience and familiarity with the context and process of research commissioning, aside from familiarity with online communication. This suggests that greater attention to process that went beyond the more technical induction offered may have been needed.

We have noted that it is widely assumed that physical access will be enhanced in CMC. This is reflected in the literature and the aims of the Programme studied here. However, the picture in practice was more complex. For example, one member with academic expertise in the theme area of his Group who was also experiencing physical disability was quite clear that being able to participate from his own computer was an advantage of the approach, yet he still did not experience the process as accessible and his participation decreased throughout so that he did not participate at all in Phase Two. This was related in part to a feeling of lack of interactivity and response, discussed below, which discouraged participation at a time when, in his view, he needed positive reinforcement to continue. In contrast, a practitioner member noted that without the virtual approach he simply would not have been able to participate, due to the inflexible nature of his clinic duties. In such cases access was about relationships between time and physical place

Access to the technology in itself did not prove to be a major barrier, since all but one member had computer and internet access, and this person used public facilities very effectively. However established patterns of using computers and online communication were important, since some had incorporated this more into their work pattern or lifestyle than others, and some members were not sufficiently 'at ease' with this mode to take advantage of its flexibility. This was more than a technical – competence and confidence – matter, since highly active members tended to be those who had incorporated computer and internet use into their everyday and working lives in such a way that regular participation, with frequent, often short log-ins, seemed relatively easy and straightforward. In contrast, some members used to a highly structured diary-approach to work participated little in practice, despite their best intentions. These tended to be members in management and policy type roles who did not lack other forms of experience or confidence to participate. This does provide a warning, however, that changes in medium or technology may, if not prepared for appropriately, substitute new for older forms of inequality of access.

The greater time that the literature suggests is needed for CMC was built effectively into the process studied here – with a total of nineteen days, structured into phases, versus two, three hour face-to-face meetings. The asynchronous process gave members time to read, consider and reflect on a comment before making their response. A number of participants described this as a valuable aspect of the approach, a point supported by Walther and Burgoon [38]. One participant, for example, described how she could read the day's contributions before leaving the work, mull them over on her journey and then post her thoughts the next morning. Not all, however, took advantage of this aspect. While participants suggested that time to think and reflect may assist less confident and assertive members in making contributions, there was little evidence from this study that this had made a difference in practice, perhaps related to the issues of interactivity and language that are discussed below.

### Interactivity

Members in both modes (virtual and face-to-face) expressed some disappointment with levels of interaction and felt a sense of working as individuals rather than as a group. This appeared to be linked to the pace, time frame and organisation of the process overall since it was not confined to the virtual mode.

Participation was higher, in both modes, in Phase One – proposal and discussion of potential topic areas. Levels of participation at this stage varied more between the groups within each mode than across them suggesting that the medium of communication was less important than other features such as the topic and the membership mix of each group and the facilitative role of the chairperson. In the Health and Regeneration group, for example, Phase One discussion was seen as 'lively' and even 'heated' in some topic areas and generated a 7,000 word transcript overall (transcripts of the discussion could be copied from the commissioning group website). Phase Two – considering research proposals – was seen as losing momentum, with more limited discussion and dialogue. Even among those who participated, some were more active than others, offering contributions and responses to others, while others simply gave their view in the manner of a statement, without any dialogue.

This suggests that the issues of 'groupness' and 'trust' raised in the literature are not particular to CMC and present a general challenge for formal decision-making groups with a limited history. Observation of the process suggests that the way time and process is managed may be of more importance to the level of interactivity than simply time per se:

And I think what the virtual committee needs to pick up is can you bring a richness into the debate? (manager member, face-to-face group, but had previously been in the virtual pilot, comparing the two modes)

On the whole, the pattern of activity in the virtual groups followed the intended process, although the number of discussion days was extended in two cases where discussion was very slow to get started. In one case, a face-to-face meeting was organised in the time gaps between Phases One and Two due to concerns within the group about momentum and interactivity of the virtual process. This suggests that participants may perceive a need to meet face-to-face, even where CMC has been established, but also that a very extended time period, for a task-focused group, may add to feelings of lack of interactivity and a loss of momentum.

In the face-to-face mode the primary problem was, nonetheless, lack of time to conduct very detailed business in a three-hour meeting. The level of formality and structure observed within the meetings was seen by members as important to achieving this and they regarded the process as effective within these limits. In contrast, in the virtual mode, which is characterised by asynchronous communication, time lags and gaps in response led to feelings of lack of dialogue which some members described as 'lonely' or even 'threatening' in the absence of cues to help explain the lack of response. Similarly, one chairperson commented on the sensitivity of tackling this in the virtual (and printed) medium:

if somebody is being quiet and not saying anything at a face-to-face group, how do you deal with that? Well you might notice that they look anxious about something and you might just say at the end of the meeting "is everything OK"? Or, you might say openly "you are being very quiet John have you got anything to say"? Well if you do all that on text it is very threatening (manager member, experience of chairing in both modes).

These feelings point to reduced social presence in CMC [7] that can have the effect of participants being self-absorbed and less likely to form impressions of their co-members.

As a result of this, and fears about being, or appearing, dominant if very active, several members reported 'reining in' their contributions. This is interesting since even though they were not perceived as dominant by other members, active participants tended to become concerned about being perceived in this way in a medium where they could not use visual or verbal cues to gauge reasons for lack or slowness of response. Similarly, in an experimental study of conflict management in virtual project teams Montoya-Weiss *et al*. [39] found that conflict was not a major problem for interaction since other participants did not interpret particular contributions as being dominant or aggressive, perhaps due to reduced social cues. While 'social richness' may not be as important for formal decision making groups of this type as in some of the more socially oriented groups discussed in the literature, the concerns expressed about interactivity and loss of momentum in the discussion point to the need for a sufficient level of interaction to maintain motivation and to give a sense of dialogue to underpin good decision making.

The provision of information about members' backgrounds – as with the virtual meeting table that was pictured in the opening screen of the groups' web pages – has been shown to be beneficial for CM groups until more sociable interactions can be established [12]. Nonetheless, a number of members saw a key potential advantage of CMC in getting beyond a previous 'closed shop' in research. Several commented that the lack of visual or audible cues reduced feelings of lack of confidence or intimidation when facing, across a table, people who seem to have more expertise and power, but as with the 'time to read, reflect and compose', there was little direct evidence that this had encouraged less confident or experienced members to participate more fully. Nonetheless, there was evidence that in the face-to-face groups members were making assumptions about each other with consumers feeling less able to contribute, certainly in the more technical aspects of assessing research proposals.

The absence of visual and verbal cues in CMC was a challenge for interaction in the virtual groups. Techniques that can be used to enhance a sense of interaction were not well developed in the site and the training provided for virtual group members focused on technical aspects of using the meeting website rather than on how to approach dialogue and decision-making within a virtual medium. Some members attempted a conversational style, and responded to others' contributions directly using first names and examples from personal experience to illustrate their points. On the whole, though, the language of contributions – being type-written – was relatively composed and formal:

Well when you are face-to-face, whatever words you come out with you have got it accompanied by non-verbal communication. All you have is words really on the web page. So you do have to be careful, you have to be much more careful! I mean you can put little smiley faces and that kind of thing but generally speaking it is words only. (chairperson, virtual group, health service manager)

There are a number of studies that show that given sufficient time CMC participants will try to achieve desired levels of immediacy through the use of symbol systems such as emoticons, the injection of humour and linguistic devices such as 'hmmm' or 'yuk' [10,16].

Observation of the face-to-face groups illustrated that these may also be general features of formal communication and decision-making in short-term groups. Indeed, this may be interpreted as appropriate to the task and nature of the group. However, the concerns expressed about levels of dialogue do suggest that a reasonable level of social interaction is required to facilitate even formal decision making. Additionally, the stated aim of wider participation in the process called for a more inclusive and less formal approach.

Discussion in the face-to-face groups initially appeared stilted and needed a lot of prompting by the chairperson. It also tended to shift during the meetings toward dominance of technical research questions and so was increasingly led by academics. Use of language was polite, reasonable, measured and members were generally supportive of each other's contributions, including non-verbal gestures such as nods of agreement. Nonetheless, members tended to follow certain themes and not to radically change views in response to others' comments. One group, with a higher proportion of consumer and voluntary sector members raised more questions about ethics, scope, perspective of proposals, as well as procedural issues and inclusiveness of the bids. The challenge for the chairperson, in either mode, was equally in drawing a rather disparate group of 'strangers' together in a very limited time span to produce clear results. In this situation, exploration and open-ness was perceived as difficult in both modes.

### Language

This theme was closely related to those of interactivity and access since, as we have noted, the ways in which language was used in each mode had a bearing on the sense of interactivity and dialogue and this, in turn, had implications for access to full participation in the commissioning process.

Some members felt that the use of formal and technical language was a barrier to participation and reduced levels of interactivity and openness. This reflects Atkinson's [39] work on public decision-making pointing to a 'linguistic market' and 'internalised self censorship', where consumers either don't possess the private language of the organisation or profession or are deterred from submitting their contribution because they anticipate that it won't count for much:

The contributions were in a language that was not everyday. It was a kind of professional lingua. Of course in a committee that was strongly academic/professional this language would be favoured. I felt I couldn't put my thoughts forward in a way that would be accepted by them (consumer member, virtual group)

For the virtual mode, these concerns may also reflect the complexity of managing, processing and producing textual messages [9] with the added difficulty of a slower rate of information exchange [41]. In this case, uncertainty about roles, ground rules, criteria and how to proceed (what Steinfeld [42] refers to as 'environmental uncertainty') seems to have resulted in the adoption by some members of a writing style that is characteristically formal and impersonal. The importance of language to the formation and effective work of CM groups is noted by Hine [10]. Rice and Love [43] found that users learn to adapt their language and style to the restrictions of the textual medium but that this takes time.

Nonetheless, face-to-face group members expressed similar concerns. As a formal group with a publicly accountable role, the work was recognised as political with a need to be professional. The lack of familiarity of the group added to this with a tendency to use indirect communication, technical language and 'politesse' [5]. Additionally, the lack of time in face-to-face meetings meant contributions had to be concise and well worked out, with little room for examples or more open discussion.

### Time

There was no indication that overall time demands differed – each mode required considerable voluntary time commitment – but patterns of use of time were different. Additionally, in the virtual groups patterns of engagement differed among members. Some only contributed on one or two days, while others continued to contribute throughout, responding directly to others. While this may reflect personal motivation and investment in the work of the group, the patterns observed in this study also matched closely the members reported ways of engaging with CMC in general and their sense of ease with the medium. Reflecting this, some used their time flexibly with frequent short visits to the site to 'see what was happening' and respond, while some made few visits and few contributions that were composed offline then posted to the site. Some members used the time delay inherent in the medium to search for and read background material, to enhance their own confidence and contribution. Those with a more flexible and 'relaxed' approach to use of time and the medium, and where this was compatible with their work, tended to be more active participants.

As noted above, the asynchronous quality of the virtual meeting was seen as having potential advantages, despite the potential for feeling lack of response, in allowing a more informed and reflective approach:

it's not you are just hearing what you want to hear. You have to read it before you can make a comment. You have to go into transmit mode to write the comment, which actually buys you time to think (manager member, experience of both modes)

This may have implications for the quality of the decisions made.

### Quality of decision-making

In both modes, members expressed some concerns about the level of discussion on which decisions were based but this did not translate into concern about the decisions per se. There was no suggestion that decision-making would have been different in an alternative mode. This is in line with the research literature that suggests that when CMC groups do reach consensus, very small differences, if any are found in the quality of decisions reached [45]. This was also confirmed by our follow-up survey of projects which had been commissioned by a virtual group at the pilot stage. The project investigators were unaware that their work had been commissioned through a novel process and did not perceive any impact on the course or outcomes of their research projects. It was notable however, that the projects commissioned reported high levels of research capacity building and impact on practice at this early stage. It is possible that these aspects were facilitated by the commissioning process adopted.

An important feature of the virtual mode was its transparency. Clear lines could be drawn between inputs, processes outputs and decisions made whereas members in face-to-face groups felt this was rather subtle, achieved but without an explicit, clear means of arriving at final decisions. Additionally, the website transcripts provided a full record of the process. The inclusion of a voting screen – although it guided rather than determined final decisions – was also seen as helpful and democratic.

## Conclusion

The evaluation showed that it was possible to conduct research commissioning effectively and accountably using a 'virtual' computer-mediated as compared to traditional face-to-face approach. The experience showed that CMC had the potential to enhance access, openness, transparency and quality of decision-making. However, there was little evidence that such potential was fully realised in this early example. It is clear from this study and from the existing literature, that the use of technology – such as the bespoke website used here – does not determine processes or outcomes. Despite the tendency in our culture to view applications of technology as a 'fix' to achieve various desirable ends, the research indicates that its effects are dependent on social relationships and the wider processes in which it is embedded [10].

In this case, other aspects beside the medium were crucial – the overall processes and structure of the programme, the time and priority given to it, the preparation and orientation of members and the approach to selection and involvement were equally important. In both modes, active chairpersons, commitment by members, knowledge of and commitment to the theme areas, a busy, motivating environment and the anticipation of future involvement were also important to the effectiveness of the process. In widening participation, further attention to issues such as patterns of language and of time and how they influence access will need consideration. Nonetheless, we suggest that the website design could be developed further to enhance accessibility, interactivity and ease of use [45].

Key advantages of the virtual mode included the ease of physical access to meetings, for groups who find it difficult to participate for a range of reasons (such as disability, caring responsibilities, fixed clinic times), reduced visual and audible status markers, greater opportunity to contribute, with time to reflect and seek more information and compose responses. The capacity to seek further information was highlighted as particularly important to ensure the decision makers were well informed and that work done does not duplicate that done elsewhere. It is very difficult for even the most knowledgeable membership to have all relevant information to hand in a live, time limited meeting. Set against these were the longer time needed for CMC, the lack of familiarity with working and communicating in virtual groups and the perception of finality, formality and inflexibility of textual messages. Additionally, there may be a time lag in individuals' and organisations' adjustment to the more flexible and open use and management of time that virtual modes of working appear to demand. The key features and potential benefits of each mode are summarised in table 5.

**Table 5:** Key points, benefits and limitations of each mode:

	**Virtual mode**	**Face-to-Face Mode**
**Key characteristics**	Communication through website Written inputs Flexible (self timed) inputs Over a time period	Face-to-face meetings Spoken inputs Inputs typically only at meeting Time limited to meeting plus some paper-based preparation
**Advantages/benefits**	Written inputs more likely to be carefully considered Time for reflection Self managed Physical presence not needed Lack of visible (audible) status markers	Generates discussion, ideas Members can check, clarify and question each other Can obtain 'soft' information and non-verbal cues Capacity for on-the-spot reflection
**Limitations/problems**	Lack of visual cues Risk of less interactivity, dialogue and group reflection	Can get skewed by powerful/dominant individuals Physical presence required
**Outcomes** **(e.g., clarity/range of research topics, quality of research proposals)**	Vignettes checked by group More opportunities to have a say Voting Process visible, can be traced	No provision to develop and check outcomes Lack of time and flexibility Lack of presence a major gap Consensus, not always clear
**Resource implications**	Website design & update Training for CMC Technical back-up Possibly higher time costs	Admin/paper distribution Arranging meetings and venues Travel costs
**Implications for individual members**	More flexible use of time Total time commitment similar to F2F mode Easier to agree time commitment with employer	Time commitment felt to be significant For many, commitment has to be agreed with employer

On balance, members were very supportive of the experiment and many would be keen to use a CM approach in future, particularly if the approach incorporates a wider range of media or a mix of modes to utilise the advantages of each – such as key face-to-face meetings with online discussion in between. CMC was also advocated as a way of involving much wider constituencies in the process than involved in this case – for example by seeking views on priorities for research from a wide range of voluntary, consumer and professional groups. Planned use of a variety of modes could be effective in terms of efficient use of time, maximising the opportunities for members to think though their contributions, widening participation by networking, drawing on wider literature and information sources and creating a rich environment that encourages participation, discussion and facilitates decision making.

It was also apparent from this study and our background work that there is relatively little current knowledge of the effectiveness of research commissioning on which to base any judgement. Indeed, there has been little discussion of what constitutes effectiveness in health research. In this study we have focused on effectiveness of the process for ensuring that decision-making is based on adequate representation, information and dialogue through from setting priority themes and topics to selecting projects for commissioning. We have also examined effectiveness in terms of outputs of research, broadly defined to include research capacity, awareness and impact as well as 'products' such as reports (see table 4) but the scope of this was limited. Examining outcomes of research commissioning in terms of impact on healthcare or health is extremely complex and would require a longer time frame and larger scale of study. There is little evidence to support the effectiveness of the 'traditional' modes of decision-making against which novel approaches must inevitably be compared. Our study tentatively suggests that the outputs of the research commissioned early in this Programme indicate that the novel approach is feasible and may bring additional benefits if managed appropriately.

## Competing interests

The author(s) declare that they have no competing interests.

## Authors' contributions

All authors participated in the design, conduct and analysis of the study and the writing of this paper. All have read and approved the final manuscript.
